# Leader Humility and Team Innovation: Investigating the Substituting Role of Task Interdependence and the Mediating Role of Team Voice Climate

**DOI:** 10.3389/fpsyg.2017.01115

**Published:** 2017-06-30

**Authors:** Wenxing Liu, Jianghua Mao, Xiao Chen

**Affiliations:** ^1^School of Business Administration, Zhongnan University of Economics and LawWuhan, China; ^2^School of Management, Huazhong University of Science and TechnologyWuhan, China

**Keywords:** leader humility, voice climate, task interdependence, team innovation

## Abstract

Leadership has been found to be linked with team innovation. Based on social information processing theory and substitutes for leadership theory, this paper examines the influence of leader humility on team innovation. Results from 90 teams showed that leader humility will enhance team innovation by fostering team voice climate. Further, task interdependence substitutes the effect of leader humility on team innovation through an indirect way via team voice climate. This study discussed the theoretical and practical implementations of these observations.

## Introduction

The link between leadership and team innovation has received much attention in literature. There is much evidence to show that team innovation can be evoked by a range of leadership approaches, such as transformational leadership (Eisenbeiss et al., 2008; Dong et al., 2016; Jiang and Chen, 2016), charismatic leadership (Paulsen et al., 2009), empowering leadership (Burpitt and Bigoness, 1997), ambidextrous leadership (Zacher and Rosing, 2015), and shared leadership (Hoch, 2013). However, knowledge about the leadership–team innovation relationship is still incomplete from several perspectives. First, most previous studies focused on the relationship between top-down leadership approaches and team innovation, while, with very few exceptions (e.g., Hoch, 2013), whether and how bottom-up leadership styles affect team innovation remains underexplored. For example, we still lack information on whether and how leader humility, one of the bottom-up leadership styles (Owens and Hekman, 2012; Chiu et al., 2016), affects team innovation. Distinct from shared leadership, leader humility is a vertical style which conveys social signals of admitting personal limitations, publicly praising followers, and displaying a high willingness to learn from others (Owens and Hekman, 2012). Although some theorists (Owens and Hekman, 2012; Nielsen et al., 2013) have called for research to investigate the relationship between leader humility and team innovation, as far as we know, no study has empirically explored this relationship. Thus, it is necessary to investigate the effect of leader humility on team innovation to reach a better understanding about leadership–team innovation relationship.

Secondly, although we can easily reach the conclusion that leadership makes a difference to innovation, drawing on substitutes for leadership theory (Kerr and Jermier, 1978), we cannot exclude the possibility that the influence of leadership on team innovation could be substituted (Mumford and Licuanan, 2004). Although scholars have tried to explore how leadership affects team innovation under varying work conditions (Eisenbeiss et al., 2008; Rank et al., 2009), it is still not enough to reach a conclusion. Since bottom-up leadership approaches (e.g., leader humility) value much about the needs of team members (Uhl-Bien, 2006; Owens and Hekman, 2012), one may arrive at inconsistent results by focusing on a specific kind of bottom-up leadership style. From this perspective, it should be useful to explore whether work conditions could act as a substitute for the influence of leader humility on team innovation.

This paper seeks to address those theoretical gaps. We use social information processing theory (SIP, Salancik and Pfeffer, 1978) and propose that leader humility positively influences team innovation by shaping a voice climate. As SIP theory posits that individuals’ attitudes and behaviors can be shaped by environmental information cues (Salancik and Pfeffer, 1978), we believe that leader humility can enhance team innovation by fostering a shared belief that speaking up is safe and efficient (i.e., *team voice climate*). We focus on voice climate because humble leaders make team members feel safe and confident to speak up, which may foster team innovation by encouraging communication about new ideas. West and Farr (1990) has identified team climate as a crucial factor influencing team innovation (Eisenbeiss et al., 2008). Meanwhile, Rego et al. (2017) has suggested future research to investigate the impact of leader humility on team effectiveness through a climate mechanism, such as a climate characterized by speaking up. By encouraging being open to new ideas, willing to learn from others and appreciating others’ strengths, leader humility can foster a voice climate that benefits team innovation (Frazier and Bowler, 2015).

Furthermore, drawing on substitutes for leadership theory (Howell and Dorfman, 1981), we propose that task interdependence would alter the influence of leader humility on team innovation. *Task interdependence* is defined as the extent to which team members depend on each other to carry out work effectively (Bachrach et al., 2006). Previous research has noted that task characteristics, such as task interdependence (Villa et al., 2003), plays a significant role in the influence process of leadership (Kerr and Jermier, 1978; Deanne and Hartog, 2001). Since task interdependence fosters self-management teams (c.f., Langfred, 2007), we believe that the impact of leader humility on team innovation via voice climate can be substituted by task interdependence.

**FIGURE 1:**
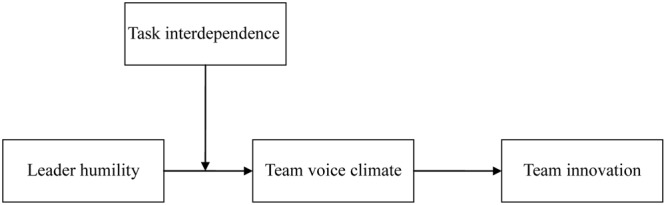
Theoretical model.

Our research aims to contribute to the literature on humility, leadership and team innovation in multiple ways. First, our research is the first to examine the relationship between leader humility and team innovation. By doing so, our knowledge about the relationship between bottom-up leadership approaches and team innovation will be increased. Second, our research clarifies how leader humility affects team innovation by revealing the mediation role of team voice climate. Third, by examining the substituting role of task interdependence on relationship between leader humility and team innovation, our research contributes to leadership literature by indicating that the effect of leadership on team innovation could be substituted. The overall theoretical model is presented in **Figure 1**.

## Theory and Hypotheses

### Leader Humility

Leader humility is defined as an interpersonal characteristic that helps leaders to better cope with social interactions by expressing a willingness to view oneself accurately, a displayed appreciation of others and their teachability (Owens et al., 2013). Conceptualized as an *interpersonal characteristic*, leader humility is behavior recognized by followers during social interactions. Previous research has identified several behavioral traits of humble leaders, such as expressing a willingness to evaluate oneself without negative or positive exaggeration demonstrating that the leader has an accurate, non-defensive, objective self-view (Tangney, 2000; Exline et al., 2004). Humble leaders appreciate the value and contributions of others (Tangney, 2002), and acknowledge the strengths of others without feeling threatened (Exline et al., 2004). Besides, they are open to new ideas, advice, and information while expressing a high interest in learning from others (Tangney, 2000).

Several studies have investigated the positive effects of leader humility across multiple levels (Owens et al., 2013, 2015; Ou et al., 2014, 2017b; Rego et al., 2017). For example, leader humility can promote followers’ job satisfaction (Owens et al., 2013; Ou et al., 2017a) and thus enhance their job engagement and performance (Owens et al., 2013). It also benefits team effectiveness by fostering collective humility (Owens and Hekman, 2016), increasing collective team psychological capital (Rego et al., 2017), and provoking team integration collaboration (Ou et al., 2014). Besides, the positive link between humble CEOs and firm performance and firm innovation has also been revealed in several recent studies (e.g., Ou et al., 2017b; Zhang et al., 2017).

Recognized as a kind of bottom-up leadership, leader humility is distinct from other leadership approaches, such as developmental leadership, servant leadership, participant leadership, and shared leadership (Owens and Hekman, 2012). For instance, in contrast to developmental leadership, which focuses exclusively on career-oriented advancement, leader humility demonstrates a transparency and mutual developmental process with followers involving many psychological aspects (Rafferty and Griffin, 2006). Although leader humility and servant leadership share some similarities, they are conceptually different in that humility could help initiate leader–follower role reversals, whereas servant leaders still play their leadership role to serve followers (Owens and Hekman, 2012). Leader humility differs from participative leadership in that it adopts specific interpersonal behaviors to promote followers’ development, whereas participative leadership emphasizes joint decision-making processes. Finally, shared leadership is a horizontal style that occurs when team members are all engaged in team leadership (Pearce, 2004), while leader humility is a vertical style that displays much greater care for the development of followers (Owens and Hekman, 2012).

### Leader Humility and Team Voice Climate

Team voice climate is conceptualized as team members’ shared beliefs about whether speaking up is effective and safe (Morrison et al., 2011). It captures employees’ understanding about their own social environment and work characteristics. For example, employees’ shared voice belief reflects their interpretations about whether their work conditions will punish them for raising inadequacy issues (Detert and Burris, 2007) and whether their teams are effective enough in implementing the ideas proposed (Gibson and Earley, 2007). Several studies have provided support for the construct validity of team voice climate by revealing its vital predictive role for both employee voice behavior (Morrison et al., 2011) and team voice behavior (Frazier and Bowler, 2015).

Schneider and Reichers (1983) argued that group climate originates from a process of collective sense-making by interacting and exchanging information with each other. More recent works on climate highlighted the importance of day-to-day social interaction for the development of shared beliefs and perceptions (Zohar and Tenne-Gazit, 2008). Thus, leadership style and leader behavior, which send strong signals to employee every day, play important roles in developing the climate (Detert and Treviño, 2010; Morrison et al., 2011). For example, Frazier and Bowler (2015) found that group perceptions of supervisor undermining negatively impact group voice climate.

Similarly, as SIP theory (Salancik and Pfeffer, 1978) posits, individual perceptions, attitudes and behaviors can be shaped by information cues, such as work requirements and expectations from the social environment (Bhave et al., 2010), while the leaders are regarded as vital sources of social information due to their direct interactions and strong influences (e.g., Boekhorst, 2015). Thus, based on SIP theory and previous group climate literatures (e.g., Zohar and Tenne-Gazit, 2008; Detert and Treviño, 2010), we propose that leader humility shapes team members’ perceptions about speaking up as humble leaders express a strong willingness to be open to new ideas and learn from others (Owens et al., 2013; Ou et al., 2017a). Generally speaking, a connection between leader humility and team voice climate is reasonable because humble leaders trigger team members’ beliefs which will enable them to characterize their work group as a safe and efficient environment to speak up by underscoring continuous delivery of the legitimacy of free communication (Owens and Hekman, 2012).

However, the unique cues for leader humility to facilitate a team voice climate can also be found if we look more specifically at each dimension of leader humility. First, humble leaders’ transparent admissions about their respective weaknesses and limitations indicate a strong willingness of leaders to accept new suggestions and implement improvement advices from employees (Owens and Hekman, 2012), which should reinforce a shared belief that speaking up is safe and necessary. Second, by appreciating team members’ strengths and contributions, humble leaders legitimize the unique strengths and expertise of team members, thus leading to a highlighted collective ability to implement useful ideas. Moreover, humble leaders also legitimize uncertainty and appreciate team members’ effort in speaking up about new ideas and changes. This makes the team members feel safe enough about sharing personally meaningful and even risky information (Nielsen et al., 2013; Chiu et al., 2016). Third, by being open to new ideas and suggestions and actively seeking feedback to improve work procedures or products (Owens et al., 2013), humble leaders can shape a shared belief that their work environment is safe enough to be able to speak up about new ideas and be efficient at implementing good suggestions. These three behavioral tendencies of humility enacted by team leaders will (1) look for, (2) recognize, (3) appreciate, and (4) implement the best ideas or suggestions, which can shape the outspoken behavior of team members. Furthermore, previous research has also indicated the positive effect of leader humility on employees’ communication and interaction about information and ideas (e.g., Owens et al., 2013; Rego et al., 2017). Taking these considerations together, we propose:

***Hypothesis 1: Leader humility is positively related to team voice climate.***

### Leader Humility and Team Innovation

Team innovation refers to the intentional introduction and application of new and useful ideas, products, processes, or procedures within a team (West and Farr, 1990). Multiple studies have already illuminated the vital role of team innovation in generating new products (Lovelace et al., 2001), improving work procedures (Miron-Spektor et al., 2011), eventually increasing competition, and fostering sustainability of organizations (Somech and Drach-Zahavy, 2013). Considering the importance of team innovation in organizations, scholars have paid much attention to how one could foster it (Hülsheger et al., 2009). For example, leaders, who exert direct influence on teams, have been regarded as a key factor contributing to team innovation (West et al., 2003; Mumford and Licuanan, 2004). For example, several empirical studies have found that both leader characteristics and leader behaviors have effect on team innovation (e.g., West et al., 2003; Eisenbeiss et al., 2008; Paulsen et al., 2009).

Seeing it as a positive leadership approach, we propose that leader humility can foster team innovation (Owens and Hekman, 2012). Owens and Hekman (2012) highlighted that humble leaders reinforce the development of themselves, their followers and teams by both setting learning or supporting programs and transparently exemplifying their developing journey through showing honesty about their own limitations and encouraging innovative ideas. Accordingly, we propose that humble leaders can promote team innovation by (1) directly launching innovation programs and (2) implicitly cultivating team members’ willingness to generate and implement new ideas. Specifically, being open to new ideas, suggestions, and solutions, humble leaders inspire team members to generate and speak up about new ideas. The teachability of humble leadership can increase the idea generation in teams by shaping a climate of voice safety and effectiveness (Pine and Gilmore, 2014). By displaying a clear awareness of their own strengths and weakness, humble leaders know better about team’s goal, team members’ characteristics and team processes, which would benefit the implementation of news ideas and procedures. Previous studies have drawn attention to the potential positive relationship between leader humility and team innovation. For example, Owens et al. (2013) found that leader humility is positively related to team learning orientation, which has been recognized as a vital predictor of team innovation (Hirst et al., 2009). Therefore, we propose the following hypothesis:

***Hypothesis 2: Leader humility is positively related to team innovation.***

### Leader Humility, Voice Climate, and Team Innovation

Several leadership researchers have suggested that, apart from the direct effect of leadership on team processes and outcomes, leadership can also influence teams in a variety of indirect ways (Graen and Uhl-Bien, 1995; Mehra et al., 2006). For example, leaders can demonstrate influence over teams by shaping a specific team climate (e.g., Schaubroeck et al., 2012). Although team voice climate has been conceptualized as a *specific climate* (Schneider, 1975; Schneider et al., 1998) with support to speaking up, several research findings suggest that team voice climate may have broader influence on team work outcomes. For example, Frazier and Bowler (2015) found that beyond its impact on team voice, team voice climate also has influence on team performance. Likewise, Frazier and Fainshmidt (2012) found that team voice climate has a positive effect on customer service performance by strengthening employees’ perceptions of psychological empowerment.

Based on SIP theory (Salancik and Pfeffer, 1978), we propose that by sending massages of being open and teachable, humble leaders enhance team members’ shared belief in speaking up, which leads in time to more team innovation. Schein (1985) argued that leaders strive to embed their values, beliefs and assumptions into member’s shared understandings, which is also called “embedding mechanisms.” Through embedding mechanisms, such as control, role modeling, coaching, workflow designs and formal statements, team members are expected to be able to shape a shared, team-level belief which, in time, will affect their attitudes and behaviors. Following these mechanisms, a leader who expresses humility will send a signal that he/she is emphasizing new and useful ideas and suggestions (Owens and Hekman, 2012; Owens et al., 2013), which will become a shared team-level belief that voice is safe in this team. By realizing that team voice safety climate is high, team members will come up with more useful ideas, which will increase the idea generation by the team (Yoshida et al., 2014). Moreover, as mentioned above, a humble leader is likely to foster the team voice climate in team, as team members believe that their new and useful ideas can be put into use. Thus they will have a greater motivation to carry out new plans as their own achievement, which in turn, should promote the idea implementation at the team level. Similarly, Yoshida et al. (2014) found that servant leaders promote team innovation by caring about team interests and development. Thus, we expected that:

***Hypothesis 3: Team voice climate mediates the relationship between leader humility and team innovation.***

### The Moderating Role of Task Interdependence

Task interdependence is conceptualized as the extent to which employees depend on other team members to carry out their own work effectively (Van der Vegt and Janssen, 2003; Bachrach et al., 2006). Task interdependence is one of the primary structural factors of teams (Campion et al., 1993; Langfred, 2007). Many researches have revealed that, when a team enjoys a high level of task interdependence, team members will be more likely to cooperate, communicate (Bachrach et al., 2006), share knowledge with others (Crawford and Haaland, 1972), and display more organizational citizen behavior (Bachrach et al., 2006). However, Langfred (2007) noted that a team’s particular structure or task design forced by certain tasks or technologies may limit the team’s ability, indicating that task interdependence can mitigate the influence of other team factors.

Besides, drawing on substitutes for leadership theory (Kerr and Jermier, 1978), leadership behaviors can be enhanced, neutralized or substituted by different situational factors (Avolio et al., 2009). Subsequent researchers have found that a variety of factors can make leadership unnecessary for followers or reduce the extent to which followers rely on their leaders, in another word, the effect of leadership could be substituted by certain contextual factors (Schriesheim, 1997). For example, it has been found that unambiguous and routine task (Deanne and Hartog, 2001), task with feedback (Kerr and Jermier, 1978), and task interdependence (Villa et al., 2003) are the key factors weakening leaders’ influences. Jermier and Kerr (1997) argued that except for reflecting strictly a moderated phenomenon, substitutes for leadership can also be introduced as a generic term to investigate its potential moderation, mediating, or main effect (Dionne et al., 2002). According to one basic premise of substitutes for leadership theory, when certain contextual factors have meaningful positive impact on employees’ behavioral outcome, these contextual factors will easily substitute the effect of leadership (Howell et al., 1986). According to literature of leader humility and task interdependence, we believe that task interdependence would temper the effect of leader humility on the team voice climate because they both have important effects on employee voice behavior.

Viewed as a bottom-up leadership approach, humble leaders demonstrate *soft power* (e.g., evaluating whether an approach is appropriate) to manage their teams rather than *hard power* (e.g., making hard decisions and being forceful when necessary, Owens and Hekman, 2012). Thus, unlike traditional, top-down leadership approaches, such as transformational leadership (Eisenbeiss et al., 2008), humble leaders may choose not to exert their influence when it is not a necessity in the situation. Thus, the influence of leader humility can be substituted more easily by other factors due to the voluntary decision of humble leaders (Owens et al., 2015). Moreover, Manz and Sims (1980) argue that self-management is a salient substitute for leadership because self-management triggers employees to instrumentally specify contingencies of self-reinforcement. These and other studies proved that high levels of task interdependence foster cooperation among team members (Podsakoff et al., 2000) and facilitate team self-management (c.f., Langfred, 2007). Thus, drawing from Manz and Sims (1980)’s theory, it is possible that a high level of task interdependence substitutes leadership by promoting self-management teams.

Several reasons could be found in supporting that task interdependence exerts similar effect on team voice climate as leader humility does, therefore substitutes the role of leader humility. First, from the perspective that employees should speak up, both task interdependence and leader humility will encourage team information exchange (Chan, 2014). To be specific, by adoring balanced information processing, humble leaders will analyze information objectively and explore other people’s opinions before making decisions, which will largely encourage information exchange (or voice behavior) between employees (Rego and Simpson, 2016). Task interdependence will also facilitate information exchange because interdependent working tasks require employees to exchange information and communicate on work issues (De Dreu, 2007). Second, from the perspective that employees will be able to speak up (Edmondson, 2003), both leader humility and task interdependent will foster formation of shared leadership, which in turn allow team members to share influence and have sense of power to speak up. Specifically, leader humility conveys leaders’ behavioral tendencies for better leader–follower interaction, which therefore legitimizes and reinforces the specific relational dynamics inherent in the formation of shared leadership (Chiu et al., 2016). Task interdependence will also work as a team structural factor that nurtures the formation of shared leadership (Hoch and Kozlowski, 2014). Third, from the perspective that employees are willing to speak up, leader humility and task interdependence will also be beneficial for cooperative climate building (Bachrach et al., 2006; Owens and Hekman, 2016). Under this circumstance, employees are more willing to speak up. Based on above statements, we believe that task interdependence will substitute the positive effect of leader humility on voice climate. Taking these considerations together, we propose:

***Hypothesis 4: Task interdependence moderates the relationship between leader humility and team voice climate such that leader humility has positive effect on team voice climate only when task interdependence is low.***

We further believe that such that task interdependence could substitute the impact of leader humility on team innovation via team voice climate. Besides, for the reason that the influence of leader humility could easily become a substitute for task interdependence, we think that, under a high level of task interdependence, humble leaders have limited influence on team voice climate, which thereupon decreases team innovation. By contrast, when task interdependence is low, team voice climate could be easily shaped by leader humility, leading to more team innovation. Thus, taking hypothesis 3 and hypothesis 4 together, we propose:

***Hypothesis 5: Task interdependence moderates the mediation effect of team voice climate pertaining to the relationship between leader humility and team innovation, such that the mediation effect is higher when task interdependence is low*.**

## Materials and Methods

### Sample and Procedure

There were no unethical behaviors in the research process, because the study did not involve human clinical trials or animal experiments, therefore, we were exempt from further ethics board approval. Ethical approval was not required for this study in accordance with the recommendations of the Science & Technology Research Office of Huazhong University of Science and Technology. Data was collected from 97 teams located in mainland China. We first contacted the team leaders and asked for their permission from a training project conducted by the local government. We assured them anonymous and strictly confidential data treatment. After securing the team leaders’ agreement for participation, we gave each leader a specific code used to make sure that the leaders and the members belong to the same team. Then, we coded the team members and asked leaders to distribute the sealed member questionnaires to all their members. We asked the managers to convey our purpose to their members that it is just a voluntary and academic research. After receiving team members’ questionnaires, we asked each leader to fill in the leader questionnaire. We sent out 97 questionnaires, from which 90 were returned (a response rate of 92.78%). Leader humility, team voice climate and task interdependence were evaluated on the basis of the responses. Team innovation was measured on the basis of responses from team leaders.

Among 90 teams, there are 36 R&D teams (40%), 24 production teams (27%), 18 sales team (20%), and 12 functional departments (13%). The average team member engaged in this research was 3.41, which makes a total of 90 team leaders and 307 team members participated in the study. For team leaders, 53% were male, with an average age of 35.41 (*SD* = 6.17) and an average work experience in present team for 6.05 years (*SD* = 4.37). For team members, 73.6% were male, with an average age of 30.89 (*SD* = 6.05) and an average work experience in present team of 5.54 years (*SD* = 5.66).

### Measurement

#### Leader Humility

We measured leader humility by using a 9-item scale developed by Owens et al. (2013). This scale is the most common measurement of leader humility (Cronbach’s alpha = 0.92). Items were rated on a 5-point scale ranging from 1 (totally disagree) to 5 (totally agree). Sample items include “My leader actively seeks feedback, even if it is critical.”

#### Team Voice Climate

We measured team voice climate using a 12-item scale developed by Morrison et al. (2011). The Cronbach’s alpha of this scale is 0.92. Respondents were asked to report the extent to which members of their team feel they are capable of effectively (or safety) to do voice behaviors proposed by Van Dyne and LePine (1998) (e.g., “develop and make recommendations concerning issues that affect the team”). Items were rated on a 5-point scale ranging from 1 (totally disagree) to 5 (totally agree).

#### Task Interdependence

We measured task interdependence by using a 4-item scale from Van Der Vegt et al. (2000) (Cronbach’s alpha = 0.80). This scale has been widely used in previous studies (Pearce and Gregersen, 1991). Items were rated on a 5-point scale ranging from 1 (totally disagree) to 5 (totally agree). Sample items include “I depend on my colleagues for the completion of my work.”

#### Team Innovation

We measured team innovation through using a 4-item scale from Drach-Zahavy and Somech (2001), which was originally developed by West and Wallace (1991). Team leaders had to indicate the extent to which the team had initiated innovations from 1 (hardly ever) to 5 (very much). Sample items include “The team initiated new procedures and methods.” The Cronbach’s alpha is 0.91.

#### Control Variable

We introduced several control variables into our analysis to minimize the effects of other exogenous variables. First, we controlled team size and team types since previous studies have found their effects on team innovation-based team processes and team innovation (see a meta-analysis from Hülsheger et al., 2009; also see Curral et al., 2001; Gajendran and Joshi, 2012). Beside, Owens and Hekman (2012) indicated that the effect of leader humility may vary across different leader gender or leader age. Thus, we controlled leader gender and leader age to better examine the effect of leader humility on team process and team innovation. Leader gender and team types were set as the dummy variable. To leader gender, “1” refers to “female,” and “0” refers to “male.” For team types, we set three dummy variables to measure four kinds of team types (i.e., R&D team, production team, sales team, and functional team).

### Analyses

We first calculated the inter-rater agreement and ICC values to make sure the variables could be aggregated to team level. The median *r*_wg_ for leader humility was 0.84, with ICC(1) and ICC(2) values were 0.52 and 0.78, respectively. The median *r*_wg_ for team voice climate was 0.75, with ICC(1) and ICC(2) values were 0.51 and 0.78, respectively. The median *r*_wg_ for task interdependence was 0.72, with ICC(1) and ICC(2) values were 0.33 and 0.63, respectively. Following the recommendation of James et al. (1984) and Schneider et al. (1998), we then aggregated leader humility, team voice climate and task interdependence to team level. Since our model did not indicate cross level effect, we used linear regression to test our model. To test the indirect effect (hypothesis 3) and conditional indirect effect (hypothesis 5), we performed bootstrapping analyses, following the suggestion of Preacher and Hayes (2004).

Since we adopted the same self-report method to measure the independent variable and the mediator, the correlation between these two variables may owe to common method bias (Dooley and Fryxell, 1999; Podsakoff et al., 2003). Thus, we then assessed the potential impact of common method bias with two additional analyses. We first conducted the confirmatory factor analysis with method factor following Widaman (1985)’s recommendation. Items were allowed to load both on theoretical constructs and on a latent common methods variance factor. Results showed that the method factor did improve model fit, however, it accounted for only a small portion (15%) of the total variance, which is almost the same or even less than the amount of method variance observed by previous studies (e.g., 27%, Williams et al., 1989; 16%, Carlson and Perrewé, 1999; 11%, Carlson and Kacmar, 2000). This result suggests that common method variance is not a pervasive problem in this study. And then we conducted the split-sample analysis, which has been suggested as one way to deal with potential team-level common method variance (Dooley and Fryxell, 1999). Then we randomly split our sample in half for each team and used one half values of the team to measure the independent variable and the other half to measure the mediator (Dooley and Fryxell, 1999). Results showed that the relationship between leader humility and team voice climate was still significant (β = 0.36, *p* < 0.01). Taken together, those analyses suggested that common method variance was not a serious threat to invalidate our findings.

## Results

### Descriptive Analysis

The means, standard deviations, and correlations for all variables are shown in **Table 1**. Leader humility was significantly related to team voice climate (*r* = 0.54, *p* < 0.001) and team innovation (*r* = 0.33, *p* < 0.01). Team voice climate was significantly related to team innovation (*r* = 0.34, *p* < 0.01).

**Table 1:** Means, standard deviations, and intercorrelations of variables.

Variable	*M*	*SD*	1	2	3	4	5	6	7	8	9
1. Leader gender	0.47	0.50									
2. Leader age	35.54	5.61	–0.10								
3. Team size	3.41	0.63	–0.05	0.23ˆ*							
4. R&D	0.50	0.50	–0.18	0.24ˆ*	0.65ˆ***						
5. Production	0.36	0.48	0.25ˆ*	–0.19	–0.37ˆ***	–0.74ˆ***					
6. Sales	0.09	0.29	–0.05	–0.11	–0.26ˆ*	–0.31ˆ**	–0.24ˆ*				
7. Leader humility	3.77	0.71	–0.02	0.15	0.05	–0.07	0.11	–0.18			
8. Voice climate	3.73	0.70	–0.01	0.07	0.04	0.01	0.09	–0.21ˆ*	0.54ˆ***		
9. Task interdependence	3.55	0.58	0.05	–0.01	0.04	–0.15	0.17	–0.10	0.37ˆ***	0.42ˆ***	
10. Team innovation	3.86	0.85	0.15	0.05	–0.07	–0.05	0.26ˆ*	–0.09	0.33ˆ**	0.34ˆ**	0.21ˆ*

*^∗^*p* < 0.05, ^∗∗^*p* < 0.01, ^∗∗∗^*p* < 0.001.*

### Measurement Model

We first used AMOS 22.0 to perform confirmatory factor analysis to verify discriminant validity of the constructs (see **Table 2**). The measurement model contained four concepts (i.e., leader humility, team voice climate, task interdependence, and team innovation) and 29 items. Results showed that the four-factor model fit the data best (χ^2^= 809.04, *df* = 371, χ^2^/*df* = 2.18, CFI = 0.91, TLI = 0.90, RMSEA = 0.06) than three-factor model (χ^2^= 1047.99, *df* = 374, χ^2^/*df* = 2.80, CFI = 0.86, TLI = 0.84, RMSEA = 0.08; Δχ^2^ = 238.95, *p* < 0.001), two-factor model (χ^2^= 1668.80, *df* = 376, χ^2^/*df* = 4.44, CFI = 0.74, TLI = 0.69, RMSEA = 0.11; Δχ^2^ = 620.81, *p* < 0.001) and one-factor model (χ^2^= 2106.07, *df* = 377, χ^2^/*df* = 5.58, CFI = 0.65, TLI = 0.60, RMSEA = 0.12; Δχ^2^ = 437.27, *p* < 0.001), confirming the discriminant validity of these constructs in our model.

**Table 2:** Confirmatory factor analyses.

Models	χ^2^	*df*	χ^2^/*df*	TLI	CFI	RMSEA	Δχ^2^	Δ*df*	*p*
Four-factor Model (LH; VL; TI; TIV)	809.04	371	2.18	0.90	0.91	0.06			
Three-factor Model (LH; VL; TI+TIV)	1047.99	374	2.80	0.84	0.86	0.08	238.95	3	<0.001
Two-factor Model (LH; VL+TI+TIV)	1668.80	376	4.44	0.69	0.74	0.11	620.81	5	<0.001
One-factor Model (LH+VL+TI+TIV)	2106.07	377	5.58	0.60	0.65	0.12	437.27	6	<0.001

*LH refers to leader humility; VL refers to team voice climate; TI refers to task interdependence; TIV refers to team innovation.*

### Hypothesis Testing

We conducted a series of linear regression models to test our hypotheses (see **Table 3**). We first entered all control variables into the model (Model 1a) and then added leader humility into the model (Model 1b). Results showed that after controlling leader gender, leader age, team size and team types, leader humility was significantly related to team voice climate (β = 0.54, *p* < 0.001), supporting hypothesis 1. Similarly, after controlling leader gender, leader age, team size and team types, leader humility was significantly related to team innovation (β = 0.40, *p* < 0.001, Model 2b), supporting hypothesis 2. Meanwhile, results also showed that team voice climate was significantly related to team innovation after controlling several controls (β = 0.32, *p* < 0.01, Model 2c). In order to test the indirect effect of leader humility on team innovation through team voice climate (hypothesis 3), we followed Preacher and Hayes (2004) recommendation to use bootstrapping method. Results from 2000 times bootstrapping showed than the indirect from leader humility to team innovation via team voice climate was 0.15, (95% confidence interval = [0.0036, 0.3817]), supporting hypothesis 3.

**Table 3:** Test of overall model.

Variable	Team voice climate			Team innovation		
	Model 1a	Model 1b	Model 1c	Model 2a	Model 2b	Model 2c
Leader gender	–0.05	–0.02	0.04	0.10	0.12	0.11
Leader age	0.05	–0.04	–0.02	0.10	0.04	0.08
Team size	0.07	–0.04	–0.17	–0.17	–0.25	–0.19
R&D	–0.17	0.17	0.33	0.75ˆ**	0.99ˆ***	0.80ˆ**
Production	–0.02	0.14	0.18	0.83ˆ***	0.95ˆ***	0.84ˆ***
Sales	–0.24	–0.04	0.05	0.31ˆ*	0.46ˆ**	0.39ˆ*
Leader humility		0.54ˆ***	0.45ˆ***		0.40ˆ***	
Task interdependence			0.21ˆ*			
Leader humility ^∗^ Task interdependence			–0.24ˆ*			
Team voice climate						0.32ˆ**
*F*	0.82	5.28ˆ***	6.10ˆ***	2.85ˆ*	5.19ˆ***	4.31ˆ***
*R*^2^	0.06	0.32	0.41	0.18	0.31	0.27
Adjusted *R*^2^	0.01	0.26	0.35	0.11	0.25	0.21
Δ *R*^2a^		0.26ˆ***	0.35ˆ**		0.14ˆ***	0.10ˆ**

*^∗^*p* < 0.05, ^∗∗^*p* < 0.01, ^∗∗∗^*p* < 0.001. All regression coefficients are standard regression coefficients. a, Model 1b and 1c was compared with Model 1a; Model 2b and 2c was compared with Model 2a.*

**FIGURE 2:**
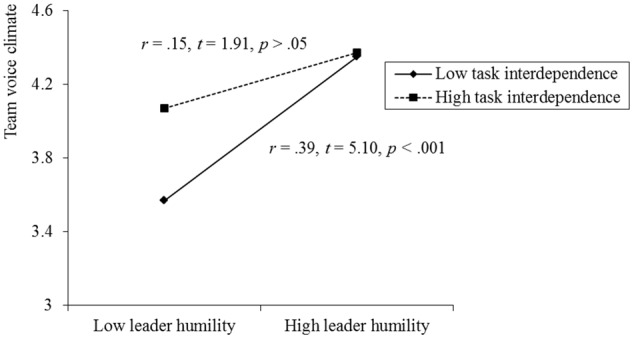
Moderating effect of task interdependence on relationship between leader humility and team voice climate.

To test moderation effect (hypothesis 4 and 5), we first conducted linear regression analysis. Results from Model 1c showed that the interaction between leader humility and task interdependence was significantly related to team voice climate (β = -0.24, *p* < 0.05). We then plotted the interaction effect and conducted the simple slope analysis (see **Figure 2**). Results showed that under low level of teak interdependence leader humility had positive effect on team voice climate (*r* = 0.39, *t* = 5.10, *p* < 0.001), while under high level of task interdependence the relationship between leader humility and team voice climate was not significant (*r* = 0.15, *t* = 1.91, *ns.*), supporting hypothesis 4. We followed Preacher and Hayes (2004) recommendation to test the conditional indirect effect (hypothesis 5). When testing conditional indirect effect, we controlled conditional effect of task interdependence on relationship between leader humility and team innovation (Preacher and Hayes, 2004; Muller et al., 2005). Results from 5000 times bootstrapping showed that the conditional direct effect is -0.39, (95% confidence interval = [-0.7291, -0.0565]). Specifically, when task interdependence is low, the indirect effect was 0.61, (95% confidence interval = [0.2583, 0.9629]), while when task interdependence is high, the indirect effect was not significant (indirect effect = 0.15, 95% confidence interval = [-0.1724, 0.4737]). Meanwhile, results showed that the moderated mediation effect (i.e., conditional indirect effect) is -0.05, (95% confidence interval = [-0.1520, -0.0003]). Specifically, when task interdependence is low, the indirect effect was 0.11, (95% confidence interval = [0.0182, 0.2505]), while when task interdependence is high, the indirect effect was not significant (indirect effect = 0.02, 95% confidence interval = [-0.1053, 0.1167]), supporting hypothesis 5.

## Discussion

The present study investigates how leader humility improves team innovation through cultivating team voice climate. In the event, we found that leader humility fosters voice climate among team members and then promotes team innovation. Furthermore, we found that task interdependence substitutes the effect of leader humility on team innovation via team voice climate. As our results showed, only under conditions of low level of task interdependence, leader humility will enhance team voice climate and then foster team innovation.

### Theoretical Implications

Our research has contributed to the literature in multiple ways. We have shown that leader humility has a significantly positive effect on team innovation by fostering a team voice climate, which increases our knowledge about the relationship between bottom-up leadership approaches and team innovation. Indeed, the term “humility” comes from Latin *humus*, which means “on the earth,” and thus leader humility means “leading from the ground” or “bottom-up leadership” (Owens and Hekman, 2012). Several studies have examined the bottom-up nature of leader humility by revealing its effect on initiating leader–follower role reversal (Owens and Hekman, 2012) and fostering self-management teams (Chiu et al., 2016). Similar with our finding that leader humility can foster team innovation, previous studies have also found that shared leadership, another bottom-up leadership style, is positively associated with team level’s innovative behavior. Meanwhile, except bottom-up leadership approaches, the relationship between top-down leadership styles and team innovation has also been investigated. For example, transformational leadership (Eisenbeiss et al., 2008) and charismatic leadership (Paulsen et al., 2009) are found to be linked with team innovation. Thus, although several scholars have distinguished between top-down and bottom-up leadership styles from multiple viewpoints, such as the source of legitimacy (Owens and Hekman, 2012) and the influence tactics (Marion and Uhl-Bien, 2002; Day and Harrison, 2007), it seems that the leadership–team innovation relation is relatively stable across different leadership styles. Thus, our research contributes to leadership literature by increasing our understanding about the relationship between leadership and team innovation.

Our research has also contributed to leader humility literature by answering the calls for examining how leader humility influences team outcomes and by clarifying the team-level mechanisms related to voice climate underlying the relationship between leader humility and team innovation. Although the significant effect of leadership on team innovation has been examined, different leadership styles emphasize different influence tactics on team innovation. For example, transformational leadership fosters team innovation by sharing the same vision and goal with team members (Dong et al., 2016; Li et al., 2016). Paulsen et al. (2009) found that charismatic leaders promote team innovation by supporting a sense of team identity and commitment, and encouraging team members to cooperate through idea articulation. However, our study showed that, without shaping a shared vision, leader humility would have a significant effect on team innovation by fostering team voice climate. Thus, our research provides evidence for DeRue’s (2011) argument about shaping a vision or a collective goal is not necessary for leadership. Moreover, although voice behavior has been receiving much attention over the last few decades (Detert and Burris, 2007; Ng and Feldman, 2013), it is not long before voice climate is regarded as a kind of team climate (Morrison et al., 2011). Although Morrison et al. (2011) proposed the concept of team voice climate and pointed to its unique value in team, how to foster team voice climate is still under discovered. Extending their work, we have explored the antecedents of team voice climate to uncover its mediating role in the relationship between leader humility and team innovation. Thus, our research both contributes to leader humility literature by revealing its team-level mechanism and expands the work of West and Wallace (1991) about the important role of team climate on team outcomes.

Further, our research has essentially answered the question whether the influence of leadership (i.e., leader humility) on teams can be substituted, which provides further empirical evidence for leadership substitute theory. Kerr and Jermier (1978) identified 13 characteristics that could act as a substitute of leadership, including c*losely knit, cohesive, interdependent work groups*. Similarly, previous research also found that self-management teams can substitute the influence of leadership on teams (Manz and Sims, 1980). Further, our research found that task interdependence substitutes the effect of leader humility on team voice climate as well as the subsequent team innovation, which provides evidence for leadership substitute theory. Taken those conclusions together, it seems that when team’s social connection between team members is strong, the influence of leader on team will decrease (Kerr and Jermier, 1978). Thus, other team characteristics reflecting the strength of connection among team members, such as the density of team social interaction or informal social network, may also act as substitutes of leadership.

Besides, our research also contributes to leader humility by casting light on whether leader humility is effective across different work conditions. Previous scholars of leader humility have emphasized the importance of specific conditions in leader humility process (Owens and Hekman, 2012; Ou et al., 2014). However, up to now, little research has explored the conditions under which condition the effect of leader humility will be strengthened or weakened (Ou et al., 2017a). Based on substitutes for leadership theory, our research has found that task interdependence can be substituted for the influence of leader humility on team innovation via team voice climate. The results of our research show that when task interdependence is high, the positive influence of leader humility on team innovation will be substituted. Thus, our research provides new knowledge for leader humility literature by examining the specific condition in which leader humility is effective.

### Limitations and Future Research

Some limitations of our research are worth emphasizing. First, this research was conducted in China, so one cannot be sure whether the findings can be generalized to Western and other cultures. For instance, in low power distance cultures (Hofstede, 1984), employees may have different understanding about leader humility and the influence of leader humility will be different. Future researchers could test our theoretical model in different cultures to achieve a more comprehensive understanding about leader humility. Second, our measure of team innovation is essentially subjective, so future researchers may use objective measurement, like numbers of patents, to measure team innovation. Third, part of our research conclusion is made based on cross-sectional data—although we have excluded the potential effect of common method bias—it is still hard for us to infer the causal relationships. Future researchers could use a longitudinal or experimental design to test the causal relationships between leader humility and team voice climate. Moreover, although leader humility can interact with task interdependence to impact teams, results also showed that leader humility is significantly related to task interdependence. This may raise an interesting question for future research to explore whether leader humility has direct effect on team members’ shared perception of task interdependence.

### Managerial Implications

In the past, a leader was perceived to be a strong-willed individual with the personality characteristics of dominance, ascendancy, and aggressiveness. By contrast, our research has found that leader humility positively impacts team climate and team innovation. Therefore, leaders should foster their humility to direct their organizations in increasingly dynamic and turbulent environment. Nielsen et al. (2013) have pointed out same exercises for leaders to cultivate humble leadership, such as embracing a vision larger than oneself, adopting a humble stance, keeping a learning diary, and practicing self-sacrifice. Following Nielsen et al. (2013)’s suggestions, companies can train leaders to express humility in focused training programs and leaders themselves should learn how to express humility. Through much effort, organizations should be able to benefit from high-quality innovations.

Although our results have shown that leader humility is positively related to team innovation, this relationship may vary in different work conditions. Our research has found that task interdependence can act as a substitute for humble leaders’ positive effect on team climate and team innovation. Thus, organizations may arrange humble leaders with some specific teams. For example, organizations may choose humble leaders to manage teams of low task interdependence in order to foster team voice climate and team innovation. Meanwhile, our results also act as a reminder to leaders that humility may not always produce positive effects. Under some specific conditions (i.e., high task interdependence), the positive effect of leader humility on some team outcomes (e.g., team innovation) may disappear.

## Conclusion

The relationship between leadership and team innovation has received much attention in literature. Our findings increase our understanding about how leader humility affects team innovation. Specifically, we have found that leader humility would enhance team innovation by fostering a voice climate. Task interdependence can act as a substitute for leader humility in fostering team voice climate and team innovation.

## Author Contributions

JM and WL designed and adopted the study, wrote the paper; XC wrote the paper.

## Conflict of Interest Statement

The authors declare that the research was conducted in the absence of any commercial or financial relationships that could be construed as a potential conflict of interest.
